# Enzyme-Catalyzed Production of Potato Galactan-Oligosaccharides and Its Optimization by Response Surface Methodology

**DOI:** 10.3390/ma12091465

**Published:** 2019-05-07

**Authors:** Mirian Angelene González-Ayón, Ángel Licea-Claveríe, José Benigno Valdez-Torres, Lorenzo A. Picos-Corrales, Rosabel Vélez-de la Rocha, Juan Carlos Contreras-Esquivel, John M. Labavitch, Josefa Adriana Sañudo-Barajas

**Affiliations:** 1 Centro de Investigación en Alimentación y Desarrollo A. C, Culiacán 80129, Sinaloa, Mexico; angelene.gonzalez.a@gmail.com (M.A.G.-A.); jvaldez@ciad.mx (J.B.V.-T.); rvelez@ciad.mx (R.V.-d.l.R.); 2 Centro de Graduados e Investigación en Química, Tecnológico Nacional de México/Instituto Tecnológico de Tijuana, Apartado Postal 1166, Tijuana, Baja California 22510, Mexico; aliceac@tectijuana.mx; 3 Facultad de Ciencias Químico Biológicas, Universidad Autónoma de Sinaloa, Culiacán 80013, Sinaloa, Mexico; lorenzo.picos.c@uas.edu.mx; 4 Facultad de Ciencias Químicas, Universidad Autónoma de Coahuila, Saltillo Coahuila 25000, Mexico; carlos.contreras@uadec.edu.mx; 5 Plant Sciences Department, University of California, Davis, CA 95616, USA; jmlabavitch@ucdavis.edu

**Keywords:** pectic galactan, enzymatic hydrolysis, endo-β-1,4-galactanase

## Abstract

This work shows an optimized enzymatic hydrolysis of high molecular weight potato galactan yielding pectic galactan-oligosaccharides (PGOs), where endo-β-1,4-galactanase (galactanase) from *Cellvibrio japonicus* and *Clostridium thermocellum* was used. For this, response surface methodology (RSM) by central composite design (CCD) was applied. The parameters varied were temperature (°C), pH, incubation time (min), and enzyme/substrate ratio (U/mg). The optimized conditions for the production of low degree of polymerization (DP) PGOs were obtained for each enzyme by spectrophotometric assay and confirmed by chromatography. The optimal conditions predicted for the use of *C. japonicus* galactanase to obtain PGOs of DP = 2 were T = 51.8 °C, pH 5, E/S = 0.508 U/mg, and t = 77.5 min. For DP = 3, they were T = 21 °C, pH 9, E/S = 0.484 U/mg, and t = 12.5 min; and for DP = 4, they were T = 21 °C, pH 5, E/S = 0.462 U/mg, and t = 12.5 min. The efficiency results were 51.3% for substrate hydrolysis. *C. thermocellum* galactanase had a lower yield (35.7%) and optimized conditions predicted for PGOs of DP = 2 were T = 60 °C, pH 5, E/S = 0.525 U/mg, and time = 148 min; DP = 3 were T = 59.7 °C, pH 5, E/S = 0.506 U/mg, and time = 12.5 min; and DP = 4, were T = 34.5 °C, pH 11, E/S = 0.525 U/mg, and time = 222.5 min. Fourier transformed infrared (FT-IR) and nuclear magnetic resonance (NMR) characterizations of PGOs are presented.

## 1. Introduction

Cancer is one of the leading causes of death worldwide [1]. Effective methods for anticancer therapies involve two different mechanisms to achieve site-specific drug delivery, passive and active targeting. Passive orientation allows a rapid vascularization of the toxic chemotherapeutic drugs through the fenestrae of the tumor tissue, whereas active targeting is considered to be a more specific system for the advanced recognition at the tumoral surface level [2]. Cancer cells express cell membrane receptors with binding affinity lectin-carbohydrate type [3]. Carbohydrates have attracted considerable interest in this field owing to their different roles in cancer progression and metastasis [4]. Specifically, modified forms of pectin polysaccharides play significant therapeutic utility against cancers and in immunomodulation process [5].

Pectins, a component of the plant’s cell wall, have three specific structural domains: (1) homogalacturonan (HG), largely assembled of a partially methyl-esterified (1-4)-linked-α-d-galacturonic acid (GalA); (2) rhamnogalacturonan I (RGI) formed by a backbone based on a repeating disaccharide of GalA and rhamnose (Rha) residues, with neutral sugars (i.e., α-l-arabinose and β-d-galactose in side chains) that are attached to the polymer backbone’s rhamnose; and (3) rhamnogalacturonan II (RGII) region, which has an HG-type backbone bearing different, relatively complex oligosaccharide branches, which contain a variety of acid and neutral sugars (i.e., rhamnose, galactose, arabinose, xylose, glucuronic acid, and 3-deoxy-d-manno-2-octulosonic acid) [6]. Plants usually contain arabinogalactans composed of (1-4)-β-galactopyranose interspersed with (1-4)-α-linked arabinopyranoses and are key components of glycoproteins with identified biological functions related to plant growth and development [7]. In general, the homo or heteropolymers have a large variety of interactions at the level of primary cell walls and play roles in the intercellular adhesion and communication, as well as serving as a reserve carbon source [8]. 

Considerations about the potential of RGI in biological tumor control applications are centered on low molecular weight oligos obtained from RGI galactan side chains, because there is evidence supporting its participation on cellular signaling being good candidates for binding to Galectin 3 (Gal3) [4]. Gal3 is a member of conserved lectins and has been considered to be diagnostic markers and/or target proteins for cancer treatment [9,10]. Gal3 has been implicated in different aspects of cancer development, including such functions as tumor cell adhesion, proliferation, differentiation, angiogenesis, cancer progression, and metastasis [10]. It is responsible for a number of diseases, specifically in overexpression in cancer and malignant hematological tumors [11,12,13]. As a result of the multivalent effect [14,15], it is thought that the binding among these linear (1-4)-β-linked-Gal PGOs may block interactions between Gal3 and other peptides, reducing their ability to promote cell adhesion and migration, and to prevent tumor growth [4,13].

**Figure 1:**
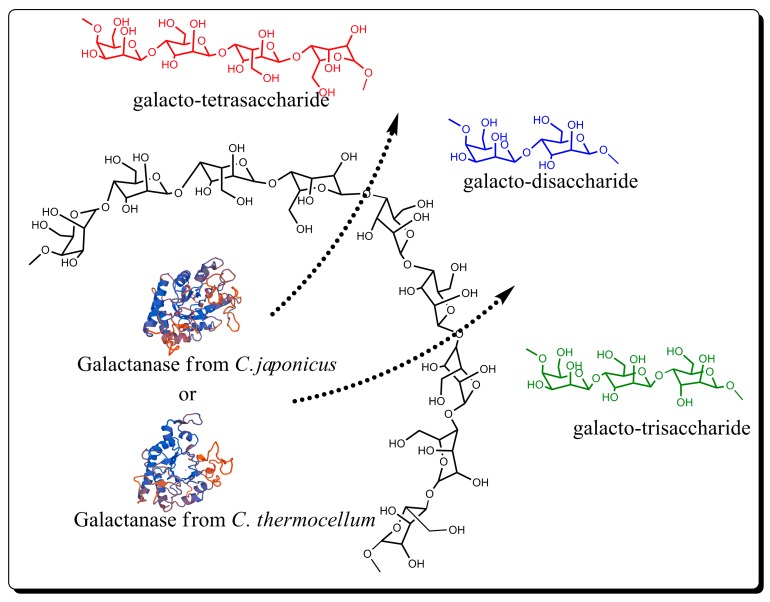
Scheme of pectic galactan-oligosaccharide production via enzymatic hydrolysis.

PGOs usually can be obtained via de-polymerization of high molecular weight pectic galactan by alkali–acid treatments [16] and enzymatic hydrolysis [17,18]. Pectin hydrolysis products used in published clinical studies involve an alkaline treatment followed by an acid treatment. On the one hand, the alkali treatment of the pectin produces the depolymerization of the RGI polysaccharide skeleton and the de-esterification of the HG regions, through β elimination reactions. On the other hand, acid treatment preferentially cleaves neutral sugars from the RGI region and leads to the release of PGOs or possibly lightly-substituted arabinogalactan oligomers [4]. The enzymatic hydrolysis using endo-1,5-α-l-arabinase, endo-1,4-β-d-galactanase (hereafter, galactanase), α-l-arabinofuranosidase, and β-d-galactosidase can be used to de-branch the RGI backbone [7,19]. The hydrolysis reaction using endo-1,4-β-d-galactanase, a member of glycosyl hydrolase family 53, according to the CAZy (Carbohydrate-active enzymes) database classification [20], should be more specific to the β-1,4-glycosidic bonds, and thus increase the yield of desired PGOs products (Figure 1). According to previous reports, PGOs with a polymerization degree (DP) between 2 and 4 have great affinity to Gal3 [5].

The pattern on depolymerization of an RGI galactan by a galactanase could vary depending of the enzyme source. A recombinant endo-galactanase from *Bifidibacterium longum*, cloned and expressed in *Escherichia coli*, had an optimal pH of 5.0 and incubation temperature of 37 °C, and produced preferentially PGOs with DP = 3, through a processive mechanism, moving toward the reducing end of the galactan chain after an initial mid- or endochain cleavage [21]. The potato pectin galactan’s (Mw ~100 kDa) hydrolysis has been investigated using endogalactanases from different origins. A novel galactanase produced by *Emericella nidulans* (anamorph *Aspergillus nidulans*) and expressed in a *Pichia pastoris* by recombination had optimal catalytic conditions at pH 5.0 and 49 °C. The enzyme was tested for its ability to catalyze potato galactan hydrolysis and, therefore, to produce PGOs as functional food ingredients, DPs were equal to 3 and 4 for reaction times of ca. 1.5 h and 30 min, respectively [17]. An endo-galactanase from *A. niger* was also used to hydrolyze potato galactan, and PGOs of DP = 11 were obtained [22]. These studies showed the feasibility of galactanase use for the production of PGOs with low molecular weight. Newly identified galactanases might be considered for optimization of the production of PGOs. However, enzymatic catalysis optimization for the production of oligosaccharides with desired DP has not been reported previously.

The aim of the present study was to examine the simultaneous influences of the main reaction parameters (temperature, pH, enzyme/substrate ratio, and reaction time) on the enzymatic hydrolysis of potato galactan. In addition, optimum conditions for the preparation of PGOs with a specific DP through the use of response surface methodology (RSM) were determined. This approach could be of interest to research groups and technologists working in the processing of potato raw materials. Also, this contribution may help other scientists studying the enzymatic hydrolysis of several biopolymers, where adequate algorithms to optimize these processes are required.

## 2. Materials and Methods

### 2.1. Materials

Pectic potato galactan (galactose/arabinose/rhamnose/galacturonic acid 82:6:3:9) and enzyme endo-1,4-β-d-galactanase from *C. japonicus* (750 U/mL) (EC 3.2.1.89) and from *C. thermocellum* (125 U/mg) (EC 3.2.1.89) were obtained from Megazyme (Bray, Co., Wicklow, Ireland); while blue dextran, raffinose, lactose, galactose standards, and other chemicals were purchased from Sigma-Aldrich (Mexico DF, Mexico). The purity of reagents was based on commercial specifications and no confirmation or further purification was performed.

### 2.2. Enzymatic Hydrolysis of Potato Pectic Galactan

The pectic galactan hydrolysis was carried out based on the variations in reaction conditions described in Table 1. Pectic galactan was used as a substrate in reaction volumes of 2 mL of sodium phosphate buffer (0.1 M) at variable pH. Reaction mixtures were held in a water bath to bring the temperature to the intended point prior to addition of galactanase. The galactan hydrolysis was measured in terms of DP average at the defined incubation times after heat enzyme denaturation at 100 °C for 10 min. The samples were freshened at room temperature (25 °C), and the PGOs were recovered by ultrafiltration (Ultra-3K filters, MWCO 3000, Amicon^®^, Merck Millipore, Billerica, MA, USA) at 5000 rpm (4696 g, in a Sorvall Legend X1R centrifuge, Thermo Fisher Scientific, Waltham, MA, USA) for 1.4 h and then freeze dried for subsequent characterization.

The relative DP was calculated by dividing the molar proportion of total sugars and reducing groups [23,24] based on anthrone [25] and 2-cyanoacetamide methods, respectively [26]. Assays were run in triplicate and evaluated in relation to a galactose standard curve.

### 2.3. Size-Exclusion Chromatography

The PGOs’ DP was confirmed by size exclusion chromatography on a Bio-Gel P2 column (Bio-Gel P2, Bio-Rad Laboratories, Hercules, CA, USA), previously conditioned with ammonium formate buffer (pH 4.5, 50 mM). The column size was 40 cm in length and 1.6 cm in diameter. Blue dextran (Mw = 2000 KDa) (2 mg), raffinose (Mw = 540.8 Da) (2 mg), lactose (360.32 Da) (2 mg), and galactose (180.16 Da) (2 mg) standards were dissolved in 2 mL ammonium formate buffer (pH 4.5, 50 mM) and the mixture was used for column calibration. The elution rate was 0.06 mL/min at 25 °C, the volume of sample charge was 1500 µL, and the fractions collected were 600 µL in size. The size distribution was determined by anthrone assay, measuring the content of total sugar over each fraction. PGOs and standard oligosaccharide elution points were compared to estimate the approximate sizes of eluted, galactanase-generated PGOs.

### 2.4. Experimental Design by RSM

RSM was used to optimize enzymatic hydrolysis of potato galactan for the production of PGOs with DP from 2 to 4. The central composite design (CCD) was used for the estimation second order response surfaces [27,28]. In this work, the factors temperature (T), pH, E/S ratio, and incubation time (t) were selected for CCD, according to previous available literature [17,29]. Levels of the factors studied and their respective coded levels (−2, lowest level; −1, lower level; 0, middle level; 1, higher level; 2, highest level) are shown in Table 1. Independent experiments were considered for each galactanase source (*C. japonicus* and *C. thermocellum*).

The arrangement for CCD was covered by 31 treatments: 2k factorial points, 2k axial points (α = 2), and 7 replicates at the center points, where k is the number of factors. The experiments were conducted for the study of linear, quadratic, and cross-product effects of the four factors each at five levels, including the center points. DP was the response variable for the combination of the independent variables. Randomized experimental runs were realized to minimize the effect of unexpected variability in the response variable. The experimental results of RSM were fitted using the following second-order polynomial equation [30]:

(1) $$Y=b_{0}+\sum_{i=1}^{k}b_{i}X_{i}+\sum_{i=1}^{k}b_{\mathrm{ii}}X_{i}^{2}+\sum^{\;}\sum_{i<j=1}^{k}b_{\mathrm{ij}}X_{i}X_{j},$$

where b_i_ is the linear coefficient, b_ii_ is the quadratic coefficient, and b_ij_ is the interaction coefficient.

The model was simplified by dropping not statistically significant terms (*p* ˃ 0.05) according to Fisher’s test for the analysis of variance (ANOVA).

The software Minitab 17 was used for the integration of the experimental design, data analysis, and model fitting. To demonstrate the effects of treatments over the generation of PGOs with different DPs, graph templates were created with the software Design Expert 7.0. A multiple non-linear regression was presented in 3D surface plots.

### 2.5. Fourier Transform Infrared Spectroscopy (FT-IR)

To analyze the chemical structure of PGOs, the spectral range from 4000 cm^−1^ to 650 cm^−1^ was registered by FT-IR (Spectrum 400, FT-IR/FT-NIR Spectrometer (Perkin Elmer, Waltham, MA, USA), including a diamond attenuated total reflection (ATR). The samples were lectured (16 scans) as dried powders.

### 2.6. Nuclear Magnetic Resonance Spectroscopy (^1^H-NMR)

The oligosaccharides were also characterized using a Bruker Avance III, 400 MHz NMR instrument (Bruker, Ettlingen, Germany). Dried powder (20 mg) was deeply dispersed in 20 mL of deuterium oxide (D_2_O) for 10 min using an ultrasonic bath, avoiding sample heating.

## 3. Results and Discussion

### 3.1. Response Surface Analysis

The influences of independent variables on PGOs production are presented in Table 2. The runs 13–16 indicate 4 of the 7 replicates at the center points, which gave exactly the same result; while the rest of the replicates are in the 3, 4, and 24 runs. The dataset shows that the potato galactan hydrolysis conditions resulted in the production of oligosaccharides with a theoretical DP between 2 and 14 galactose residues. In general, the depolymerization was more effective in the experiments with galactanase from *C. japonicus* relative to the *C. termocellum*; this is reflected in the lower DP for the *C. japonicus* PGOs.

Table 3 shows the results for experiments where galactanase from *C. japonicus* was used. The results obtained when the *C. thermocellum* enzyme was used are shown in Table 4. The value of *p* ˃ F of the model was considerably less than 0.05, indicating that the model provided a significant and useful prediction at the 0.05 level. The result of the ANOVA shows only the factors with *p* values ˂ 0.05. For both experiments using the *C. japonicus* and *C. thermocellum* enzyme, the final response surface regression model was fitted with the second order polynomial Equations (2) and (3), respectively. For the *C. japonicus* enzyme, the model had an acceptable coefficient, *R*^2^ = 0.853, indicating that more than 85% of the variation could be explained by the independent factors. Therefore, the predicted values and the experimental results in terms of PGOs generation are in good concordance, meaning that this model will be useful for the purpose of optimization. For the *C. thermocellum* galactanase, the model has a coefficient of determination, *R*^2^ = 0.902. These results may also serve to identify the best conditions for obtaining oligosaccharides with high reducing sugar content or low DP.

(2) $$Y=0.24182-0.02826X_{1}-0.044223X_{2}+0.02359X_{3}+0.01402X_{4}-0.02794X_{1}^{2}\;-0.01025X_{2}^{2}-0.02685X_{1}X_{2}$$

(3) $$Y=0.2422+0.001625X_{1}-0.0574X_{2}+0.1310X_{3}+0.00024X_{4}+0.002894X_{2}^{2}$$

where Y is the polymerization degree, X_1_ is reaction temperature, X_2_ is pH, X_3_ is enzyme/substrate ratio, and X_4_ is the reaction/incubation time.

Table 3 and Table 4 list the *F* test, the corresponding *p* values, along with the parameter estimated. The smaller the *p* value, the larger the significance of the corresponding coefficient [31]. The significance of each coefficient was determined using the *p* value as reference. Values less than 0.05 indicated that there is statistical significance for a model term [32].

Afterward Table 3 and the model Equation (2), it could be concluded that for galactanase from *C. japonicus*, the lineal terms temperature, pH, E/S ratio, and incubation time (X_1_, X_2_, X^3^, and X_4_); the quadratic terms X_1_^2^, X_2_^2^; and the interaction term X_1_X_2_ could all significantly affect the degree of polymerization (*p* ˂ 0.05). However, between these statistically significant variables, the pH (X_2_) was the most important factor influencing the extent of hydrolysis. This effect was followed by T^2^ (X_1_^2^), T (X_1_), E/S (X_3_), T * pH (X_1_X_2_), time (X_4_), and pH^2^ (X_2_^2^). In contrast, E/S^2^ (X_3_^2^), T * E/S (X_1_X_3_), and pH * E/S (X_2_X_3_) had no significant effects on the hydrolysis reaction and, therefore, would not be useful for influencing oligosaccharide degrees of polymerization.

Likewise, the ANOVA for reactions employing the *C. thermocellum* galactanase show that only the lineal terms and pH^2^ (X_2_^2^) are statistically significant. As can be seen in Table 4 and Equation (3), temperature (X_1_), pH (X_2_), E/S (X_3_), reaction time (X_4_), and the quadratic term X_2_^2^ could have an impact on the content of reducing sugars of products.

**Figure 2:**
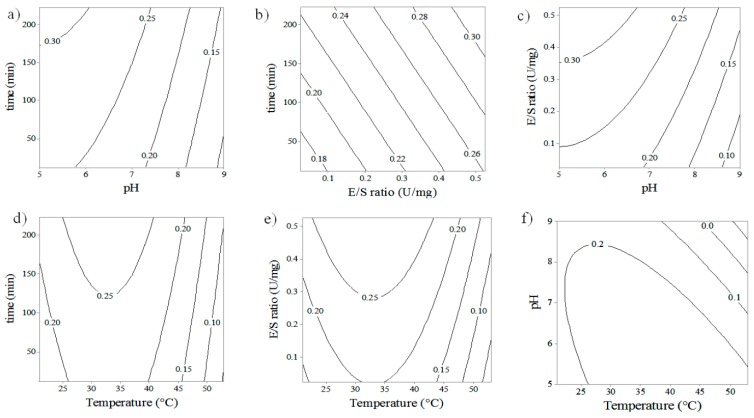
Contour plots depicting the interactive effect of two factors over the liberation of reducing group from hydrolysis of potato galactan by *C. japonicus* galactanase. (**a**) pH * time; (**b**) E/S * time; (**c**) pH * E/S; (**d**) temperature * time; (**e**) temperature * E/S; (**f**) temperature * pH.

**Figure 3:**
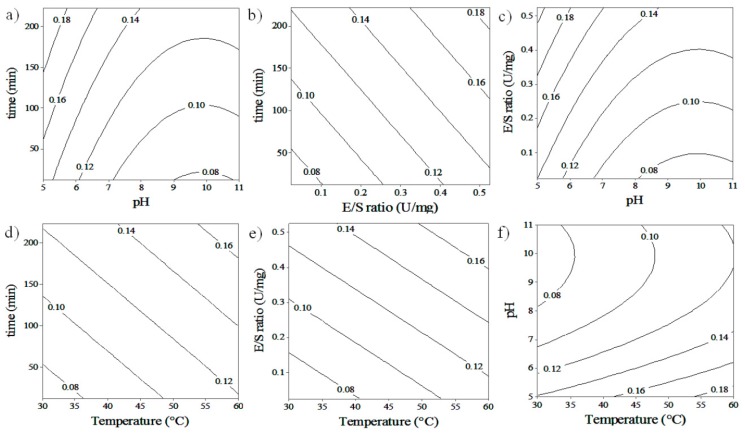
Contour plots depicting the interactive effect of two factors over the liberation of reducing group from hydrolysis of potato galactan by *C. thermocellum* galactanase. (**a**) pH * time; (**b**) E/S * time; (**c**) pH * E/S; (**d**) temperature * time; (**e**) temperature * E/S; (**f**) temperature * pH.

Contour plots of hydrolysis reactions were made in order to analyze the relationship between the different variables on PGOs’ size, with the ultimate goal of obtaining the best reaction conditions for producing PGOs with a desired content of reducing sugars, implying a desired degree of polymerization. While the interaction of some factors was analyzed, others were fixed at their center points. The interaction between pH and time produces a higher content of reducing sugars (i.e., lower DP) on PGOs at a higher time of reaction and lower pH; this behavior is observed in experiments in which both endogalactanases were used (Figure 2a or Figure 3a). On the other hand, the interaction between E/S ratio and reaction time demonstrates a slight reduction of DP for the enzyme from *C. japonicus* when the time is varied at certain E/S ratios. For the enzyme from *C. thermocellum*, a chain reduction is observed with the increase of the E/S ratio and reaction time.

Figure 2a–c and Figure 3a–c show the influence of the pH and the E/S ratio on the reducing sugar content on products. For *C. japonicus*, a decrease of RS is observed when the pH is decreased from 9.0 to 5.0 at an E/S ratio of 0.25 U/mg, and a slight increase of RS appears once the E/S ratio increases from 0.15 up to 0.4 U/mg. For *C. thermocellum*, a similar pH effect on DP is observed when the pH changes from 11.0 to 5.0, and the RS content increases slightly when the E/S ratio is increased from 0.15 to 0.4 U/mg. The pH effect on the catalysis reactions is mainly attributed to the modification of the conformational structure of the enzyme [27], based on the idea that the native conformation is preferred for maximum enzyme activity. In Figure 2a–c and Figure 3a–c, it can be seen that during the hydrolysis reaction, in spite of an increase in the E/S ratio from 0.1 to 0.5 U/mg, a slight increase in product reducing sugar content (i.e., decrease DP) was observed. These results can be explained by potentially more enzyme availability to react with and form an enzyme–substrate complex with the substrate. Furthermore, this might also imply that increasing the enzyme concentration leads to a point where the reaction rate becomes constant. This is effective if the concentration of substrate molecules becomes the limiting factor, that is, all the substrate molecules were interacting with an enzyme, so that all enzyme active sites were occupied with the substrate molecules [33].

In the case of *C. japonicus* enzyme galactanase (Figure 2d–f), the liberation of reducing sugars increased from 20 to 35 °C and presented a behaviour of reduction in reducing group liberation from 35 to 50 °C, showing quadratic behavior. The experiments with the *C. thermocellum* enzyme (Figure 3d–f) showed a lower content of reducing sugar or higher PGOs’ size when the temperature decreased from 60 to 30 °C. The same behavior was observed when the pH, E/S ratio, and incubation time were changed simultaneously with the temperature. According to the manufacturer, the physicochemical properties for *C. japonicus* galactanase are suitable at a pH range from 6 to 8 units, T of 40 °C, whereas *C. thermocellum* is presented with optimum conditions at pH 4.5 and T of 60 °C. The explanation of this behaviour is challenging, however, thermodynamic is often of higher significance than theorical pronostics when the kinetic is affected by several variants such as pH, [E]/[S] ratio, and incubation time. Temperature by itself has two different effects on catalysis reaction when it is established in a range. First, it can alter the enzyme–substrate interaction frequency [34,35], increasing the hydrolysis of glycosidic bonds, leading to an increase of enzyme activity and producing smaller PGOs. The collision must have enough energy for the particles to react (activation energy). On the other hand, modifying temperature and/or pH of the reaction can influence the enzyme´s activity by changing its conformational structure [36] and lead to the enzyme´s inability to recognize, bind to, and catalyze Gal–Gal bonds in the substrate, perhaps leading to the production of fewer PGO products of larger size and, preferentially, of less value in terms of tumor-targeting activity. Our assays confirmed the functional activity of the enzymes at the experimental conditions provided, however, conditions out of range of testing might eventually lead to enzyme inactivation.

### 3.2. Confirmation and Validation Experiments

**Figure 4:**
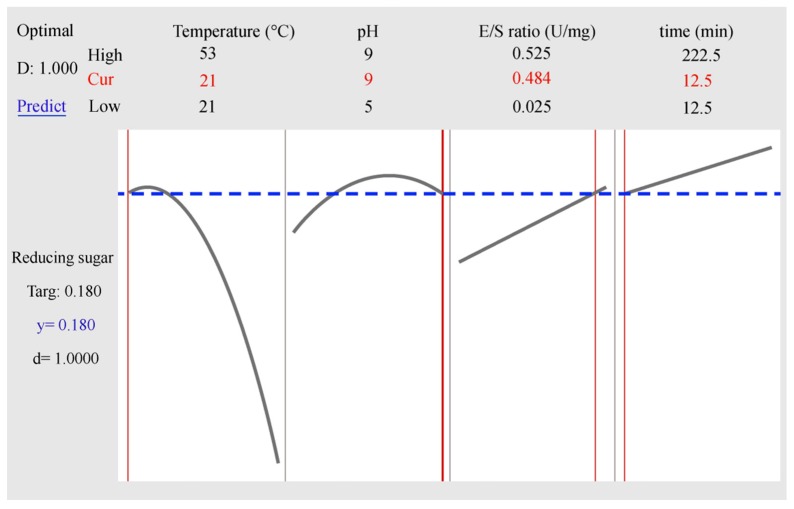
Optimization results by MINITAB17 for PGOs with DP around 3, using the *C. japonicus* enzyme.

The equations obtained from the experimental data were used to predict the average polymerization degree of PGOs as temperature, pH, E/S ratio, and hydrolysis time were varied in the range of the experimental design. As an example, Figure 4 shows the best conditions for the enzymatic reaction to obtain 0.18 μmol of reducing sugar corresponding to PGOs with DP = 3 using the *C. japonicus* galactanase, when T = 21 °C, pH 9, E/S ratio = 0.484 U/mg, and with 12.5 min reaction time. The two curves presented in the figure represent the maximum points for the hydrolysis at the optimizing zone for temperature and pH, while the straight lines represent the best conditions for E/S ratio and reaction time; however, the combination of these four parameters allows for the prediction of the average PD of PGOs with the highest grade desired (d = 1).

In order to confirm the validity of the suggested mathematical model, six confirmation experiments were done with three independent replicates under the conditions obtained by the optimization of the regression model (Table 5). The predicted and observed values for PGO DP were compared. Under these conditions, the average DPs of products are in total accordance with the predicted values. Therefore, the estimated empirical models were convenient, and the RSM method seems to be a useful approach to predict the best reaction conditions for the production of PGOs with the desirable polymerization degree.

### 3.3. Size-Exclusion Chromatography (SEC)

**Figure 5:**
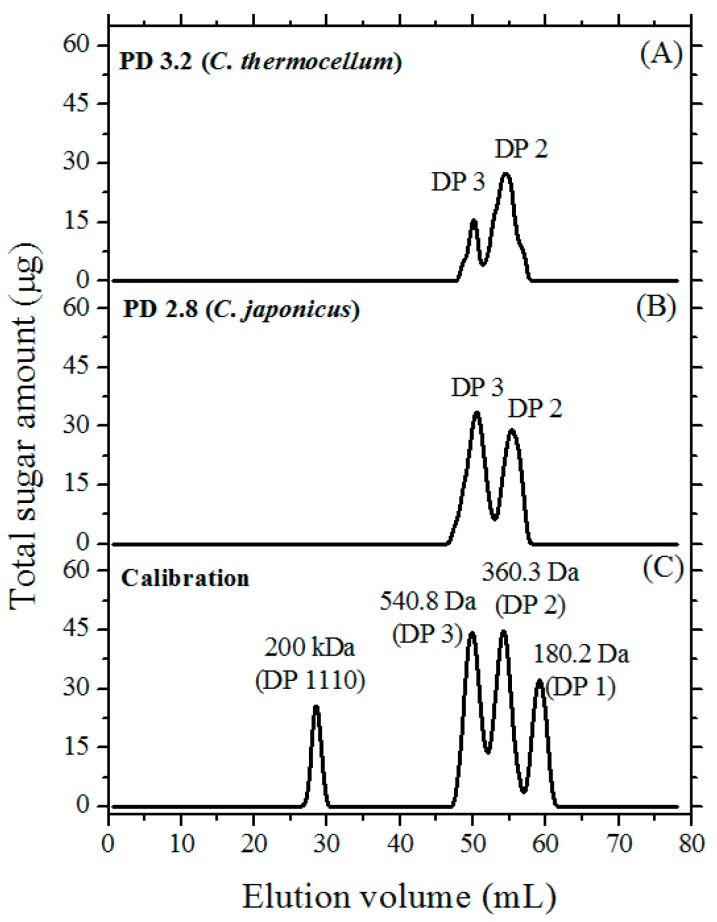
Size-exclusion chromatography (Bio-Gel P2) profiles of reactions optimized for generation of PGOs with DP 2.8–3.2. (**A**) galactanase from *C. thermocellum*; (**B**) galactanase from *C. japonicus*; and (**C**) calibration profile using known mono-, di-, and tri-saccharide standards and blue dextran (high molecular weight). Bio-Gel P2 column fractions (0.6 mL) were collected and assayed for total sugar content using the anthrone method.

The PGOs obtained using the optimized methodology were subjected to SEC to examine the distribution in PGOs’ DPs. A nicely separated distribution of peaks was observed (Figure 5). The profile corresponded to a mixture of disaccharides and trisaccharides. The amount of trisaccharides in the collected PGOs was around 12.9%, while disaccharides accounted for 22.8% in experiments with the *C. thermocellum* enzyme. In this case, the overall PGOs’ yield was 35.7%. On the other hand, the *C. japonicus* enzyme yielded 27.5% of trisaccharides and 23.8% of disaccharides for an overall production of 51.3%. No PGOs with larger DPs were detected, indicating that the galactan substrate was completely hydrolyzed.

### 3.4. FT-IR and NMR Characterization

**Figure 6:**
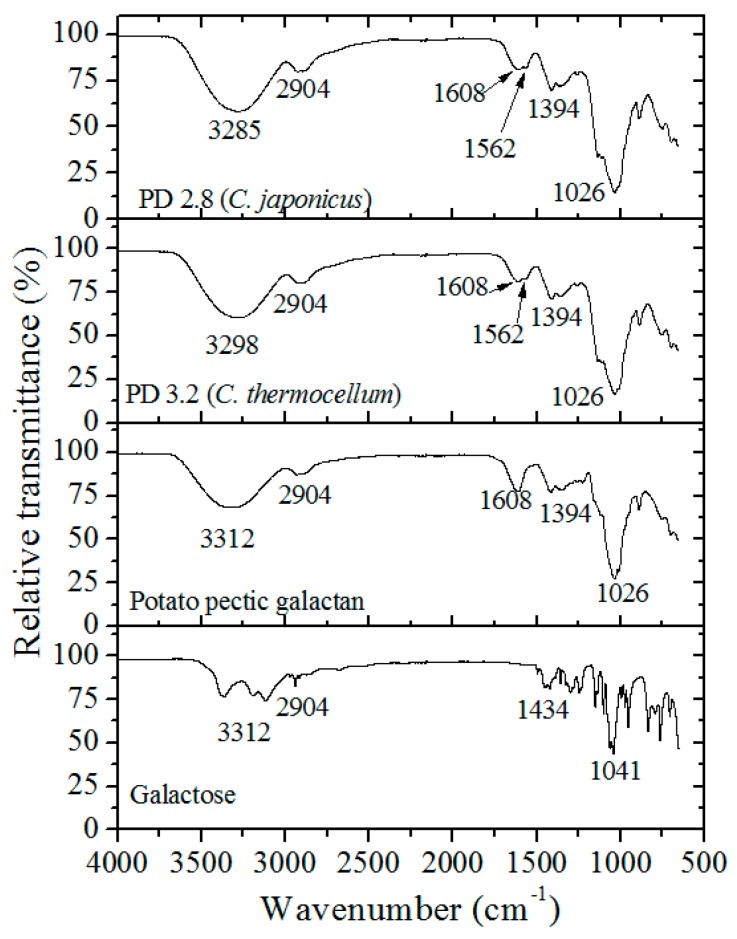
Fourier transform infrared (FT-IR) spectra of galactose standard, potato galactan, and PGOs produced by *C. japonicus* and *C. thermocellum* enzyme.

The PGOs’ chemical structure was contrasted with the spectra of galactose standard, potato galactan, PGOs, and optimized PGOs generated by the *C. japonicus* enzyme (Figure 6). Spectra perfectly contain the distinctive bands of galactose units: the band around 3298 cm^−1^ is attributed to –OH stretching, the band around 2904 cm^−1^ is attributed to the –CH_2_–; and the band around 1608 cm^−1^ is attributed to carboxylate (COO^−^) from the uronic acid of pectic substances. The peak at 1562 cm^−1^ is related to a signal from bound water [37], that at 1394 cm^−1^ corresponds to –CH, and that at 1036 cm^−1^ corresponds to ‒C–OH of alcoholic groups and carboxylic acids [38].

**Figure 7:**
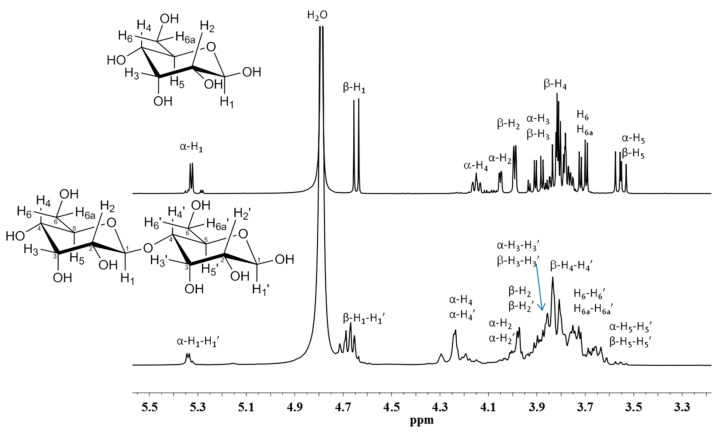
Proton nuclear magnetic resonance (^1^H-NMR) spectra of galactose standard and PGOs produced via enzymatic hydrolysis with *C. japonicus* enzyme.

The purity of the PGOs produced was evaluated by proton nuclear magnetic resonance (^1^H-NMR). Figure 7 shows the ^1^H-NMR spectra for galactose standard and PGOs produced via enzymatic hydrolysis. The signals at 5.3 and 4.7 ppm are attributed to the hydrogen of anomeric carbon (H_1_) in the α and β position, respectively. Hydrogen (H_2_) was attributed to the signals at 4.0 and 3.95 ppm; hydrogen (H_3_) was assigned to the signal at 3.85 ppm; hydrogen (H_4_) was at 4.2 and 3.8; and hydrogen (H_5_ and H_6_) were at 3.5 and 3.7 ppm, respectively. Basically, the same signals were shown by these PGOs. The broad signals were attributed to the interactions between the –OH groups in the molecule.

## 4. Conclusions

Controlled enzymatic degradation of pectic galactan resulted in PGOs’ samples varying in polymerization degree, all of which were lower than four galactose residues. The catalysis was influenced by temperature, pH, enzyme/substrate ratio, and incubation time. The model validation provided an appropriate agreement between the experimental results and the predicted response (*R*^2^ = 0.853 for galactanase from *C. japonicus* and *R*^2^ = 0.902 from *C. thermocellum*).

Endo-galactanase from *C. japonicus* gave a higher yield (51.3%) in terms of PGOs’ production relative to the enzyme from *C. thermocellum* (35.7%). Thus, the *C. japonicus* enzyme has been regarded as the most promising candidate for the production at scale of galactose-rich PGOs with PD < 4 for its potential application for recognition to cancer cells (i.e., as inhibitor of pro-metastasic Galectin 3). Further studies are needed to confirm the successful interaction between PGOs obtained and the protein Galectin 3 at conditions that mimic physiological conditions.

## Figures and Tables

**Table 1:** Ranks of the levels designed for the experimental design (CCD). The levels of T and pH were established according to the technical data of enzyme stability from the manufacturer Megazyme.

Variables	Symbols	Levels				
		*C. japonicus*/*C. thermocellum*				
		–2 ^a^	–1	0	1	2 ^a^
T ^b^	X1	21/30	29/40	37/50	45/60	53/70
pH	X2	5/3	6/5	7/7	8/9	9/11
E/S ^c^	X3	0.025	0.15	0.275	0.4	0.525
t ^d^	X4	12.5	65	117.5	170	222.5

^a^ axial point (α = 2); ^b^ incubation temperature (°C); ^c^ enzyme/substrate ratio (U/mg); ^d^ incubation time (min).

**Table 2:** Experimental design.

Run	Coded Level of Variable				Reducing Sugar		DP ^f^	
	T ^a^	pH	E/S ^b^	T ^c^	*C.j*. ^d^	*C.t*. ^e^	*C.j*. ^d^	*C.t*. ^e^
1	−1	−1	−1	−1	0.2048	0.1427	2.7	4.1
2	2	0	0	0	0.0383	0.1421	14.2	4.1
3	0	0	0	0	0.2131	0.1166	2.6	5.0
4	0	0	0	0	0.2670	0.1205	2.0	4.9
5	1	−1	−1	−1	0.2509	0.1599	2.2	3.7
6	1	1	−1	−1	0.0522	0.0883	10.4	6.6
7	1	−1	−1	1	0.2359	0.1998	2.3	2.9
8	−1	−1	1	−1	0.2392	0.146	2.3	4.0
9	−1	−1	1	1	0.2864	0.1743	1.9	3.4
10	1	1	1	1	0.1371	0.1349	4.0	4.3
11	0	2	0	0	0.1726	0.1132	3.2	5.2
12	0	0	2	0	0.2792	0.1682	2.0	3.5
13–16	0	0	0	0	0.2409	0.1427	2.3	4.1
17	−1	1	−1	−1	0.1349	0.0644	4.0	9.1
18	−1	1	1	1	0.2243	0.1277	2.4	4.6
19	−1	−1	−1	1	0.2548	0.0816	2.1	7.2
20	0	0	−2	0	0.1837	0.0788	3.0	7.4
21	0	0	0	2	0.2537	0.1482	2.1	4.0
22	0	0	0	−2	0.1993	0.101	2.7	5.8
23	1	1	1	−1	0.1282	0.1299	4.2	4.5
24	0	0	0	0	0.2720	0.1610	2.0	3.6
25	−1	1	−1	1	0.1965	0.086	2.8	6.8
26	0	−2	0	0	0.2415	0.0955	2.3	6.1
27	1	1	−1	1	0.0866	0.1138	6.3	5.2
28	−2	0	0	0	0.2342	0.1044	2.3	5.6
29	1	−1	1	−1	0.2737	0.1843	2.0	3.2
30	1	−1	1	1	0.2964	0.232	1.8	2.5
31	−1	1	1	−1	0.2065	0.0822	2.6	7.1

^a^ incubation temperature (°C); ^b^ enzyme/substrate ratio (U/mg); ^c^ incubation time (min); ^d^ galactanase from *C. japonicus* (*C.j.*), total sugar = 0.5447967 μmol; ^e^ galactanase from *C. thermocellum* (*C.t.*), total sugar = 0.58664211 μmol; ^f^ degree of polymerization (DP) estimated by the ratio total sugar/reducing sugar.

**Table 3:** Analysis of variance (ANOVA) for the quadratic polynomial model for level optimization of pectic galactan-oligosaccharides (PGOs) using endo-β-1,4-galactanase from *C. japonicus*.

Source	Adj. Sum of Squares	Degree of Freedom	Adj. Mean Square	F Value	*p* Value
Model	0.116164	7	0.016595	19.01	0.000
X_1_-Temp	0.019171	1	0.019171	21.97	0.000
X_2_-pH	0.042805	1	0.042805	49.05	0.000
X_3_-E/S	0.013356	1	0.013356	15.30	0.001
X_4_-time	0.004715	1	0.004715	5.40	0.029
X_1_^2^	0.022792	1	0.022792	26.12	0.000
X_2_^2^	0.003066	1	0.003066	3.51	0.074
X_1×2_	0.011536	1	0.011536	13.22	0.001
Error	0.020073	23	0.000873		
Lack of fit	0.017176	17	0.001010	2.09	0.184
Pure error	0.002897	6	0.000483		
Total	0.136237	30			

**Table 4:** ANOVA for the lineal model for level optimization of PGOs using *C. thermocellum* endogalactanase.

Source	Adj. Sum of Squares	Degree of Freedom	Adj. Mean Square	F Value	*p* Value
Model	0.035948	5	0.007190	42.32	0.000
X1-Temp	0.005213	1	0.005213	30.69	0.000
X_2_-pH	0.019945	1	0.019945	117.41	0.000
X_3_-E/S	0.006435	1	0.006435	37.88	0.000
X_4_-time	0.003940	1	0.003940	23.19	0.000
X_2_^2^	0.002132	1	0.002132	12.55	0.002
Error	0.003907	23	0.000170		
Lack of fit	0.002279	17	0.000134	0.49	0.882
Pure error	0.001629	6	0.000271		
Total	0.039855	28			

**Table 5:** Validation of optimal conditions for the production of PGOs with DPs of 2, 3, and 4.

Endo-1,4-β-galactanase	d *	T (°C)	pH	E/S (U/mg)	Time (min)	RS_pred_	TS (μmol)	RS (μmol)	DP_cal_
*Cellvibrio* *japonicus*	1	51.8	5	0.508	77.5	0.27	0.478	0.254 ± 0.016	1.9
	1	21	9	0.484	12.5	0.18	0.475	0.173 ± 0.007	2.8
	1	21	5	0.462	12.5	0.13	0.579	0.137 ± 0.007	4.2
*Clostridium* *thermocellum*	1	60	5	0.525	148	0.23	0.576	0.274 ± 0.044	2.1
	1	59.7	5	0.506	12.5	0.19	0.597	0.184 ± 0.006	3.2
	1	34.5	11	0.525	222.5	0.14	0.617	0.151 ± 0.011	4.1

* d = acceptability of experiment (0 ≤ d ≤ 1); TS = total sugar (μmol); RS = reducing sugars (μmol); RS_pred_ = RS predicted by Minitab program; DP_cal_ = TS/RS.
